# Two-Component System Response Regulator *ompR* Regulates Mussel Settlement through Exopolysaccharides

**DOI:** 10.3390/ijms24087474

**Published:** 2023-04-18

**Authors:** Wei Ma, Xiaoyu Wang, Wen Zhang, Xiaomeng Hu, Jin-Long Yang, Xiao Liang

**Affiliations:** 1 International Research Center for Marine Biosciences, Ministry of Science and Technology, Shanghai Ocean University, Shanghai 201306, China; 2 Shanghai Collaborative Innovation Center for Cultivating Elite Breeds and Green-Culture of Aquaculture Animals, Shanghai 201306, China

**Keywords:** *Pseudoalteromonas marina*, *ompR* gene, OmpW, biofilm, *Mytilus coruscus*, settlement, exopolysaccharide

## Abstract

The outer membrane protein (OMP) is a kind of biofilm matrix component that widely exists in Gram-negative bacteria. However, the mechanism of OMP involved in the settlement of molluscs is still unclear. In this study, the mussel *Mytilus coruscus* was selected as a model to explore the function of *ompR*, a two-component system response regulator, on *Pseudoalteromonas marina* biofilm-forming capacity and the mussel settlement. The motility of the Δ*ompR* strain was increased, the biofilm-forming capacity was decreased, and the inducing activity of the Δ*ompR* biofilms in plantigrades decreased significantly (*p* < 0.05). The extracellular α-polysaccharide and β-polysaccharide of the Δ*ompR* strain decreased by 57.27% and 62.63%, respectively. The inactivation of the *ompR* gene decreased the *ompW* gene expression and had no impact on *envZ* expression or c-di-GMP levels. Adding recombinant OmpW protein caused the recovery of biofilm-inducing activities, accompanied by the upregulation of exopolysaccharides. The findings deepen the understanding of the regulatory mechanism of bacterial two-component systems and the settlement of benthic animals.

## 1. Introduction

*Mytilus coruscus* belongs to Mytillidae, which is a typical large fouling organism, and one of the commercial bivalve molluscs [1,2]. In the life cycle of most benthic invertebrates, the process of settlement is necessary for larvae to grow into adults [3]. Unraveling the settlement mechanism is important for developing environment-friendly macrofouling techniques and artificial breeding in marine aquaculture. In the marine environment, a huge number of bacteria exist in the form of biofilms, which coordinate the settlement of benthic animals including mussels [4,5,6].

Biofilms are composed of bacteria and matrix, which can attach to the surface of the base and grow [7,8]. In most bacterial biofilms, the biomass of extracellular polymeric substances (EPS) is much higher than that of bacteria, and the EPS include exopolysaccharides, exoproteins, exolipids, etc., which contribute greatly to the formation of biofilms [9,10]. It was proved that some particular bacterial strains in biofilms can promote benthic invertebrates to settle [11,12,13,14]. 

Gram-negative bacteria are the dominant bacteria in marine bacteria, which are protected by inner and outer membranes [15]. The outer membrane is a selective barrier for substances to enter cells [16], and nutrients cannot directly enter and exit the outer membrane. The outer layer of cells is the outer membrane protein (OMP), which can input nutrients and transform signals from the external environment [17]. Among them, OMPs have strong immunogenicity [18,19], and can mediate intercellular adhesion [20] and the interaction between bacteria and object surfaces, thus forming mature biofilms [21]. In many Gram-negative bacteria, it was found that OmpR can regulate the pore protein of the outer membrane [22]. As a significant global regulatory factor, OmpR can regulate a variety of physiological activities of bacteria, including the expression of outer membrane porin and virulence factors, the formation of bacterial flagella, as well as bacterial motility and chemotaxis [23,24]. The EnvZ/OmpR system of *Escherichia coli* is a widely studied two-component regulatory system that mediates signal transduction and the expression of outer membrane proteins, as well as biofilm formation [25,26,27]. In *Cronobacter sakazakii*, it was found that the biofilm formation ability of the *ompW* gene deletion mutant was increased in response to environmental stress [28]. The outer membrane protein OmpW is an elongated barrel that spans the inner and outer membranes, which can protect bacteria by resisting adverse environmental pressures [29].

*Pseudoalteromonas marina* is a Gram-negative bacterium widely present in the marine environment [30]. *P. marina* can secrete abundant EPS to form marine biofilms [6,31]. Previous studies have shown that *Pseudoalteromonas* can produce many biologically active substances [32] and induce the settlement of benthic invertebrates [13,33,34,35]. Deleting the *fliP* gene of *P. marina* brought about an increase in exoprotein, which inhibited the settlement of mussels [36]. Deleting the *01,912* genes of *P. marina* increased the colanic acid and exopolysaccharide levels, thus promoting the settlement of mussels [6]. OmpW, a prominent OMP of EPS [29,37], has been proven to regulate biofilm formation in other bacteria [28,38]. The interaction between OmpW and biofilm and how the outer membrane regulator impact OmpW remains unclear. 

This study explored the relationships between OMP regulator gene *ompR*, OmpW, biofilm formation, and mussel settlement by constructing a mutant strain of a two-component system response regulator. Here, *ompR* gene knockout in *P. marina* was used to (1) analyze the effect on the ability of bacterial growth and biofilm formation; (2) confirm the impacts on inducing mussel settlement activity; and (3) detect the expression difference in the *ompW* gene and explain the relationship between *ompR*, *ompW*, EPS, and settlement.

## 2. Results

### 2.1. Colony Morphology, Growth, Swimming Motility, and Biofilm-Forming Ability

**Figure 1:**
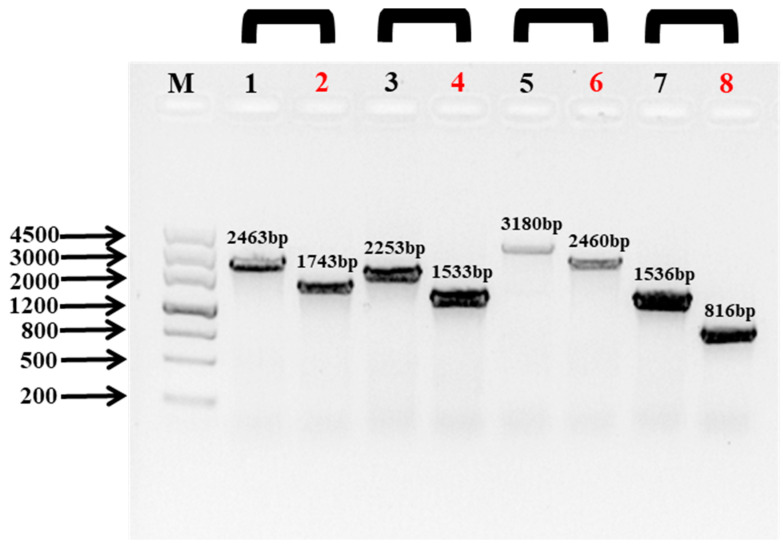
The deletion of the *ompR* gene was confirmed by PCR. M: DNA MakerIII; Wild-type in lanes 1, 3, 5, and 7; Δ*ompR* in lanes 2, 4, 6, and 8. Lane 1 (*ompR*-SF/LR, 2463 bp), lane 2 (Δ*ompR*-SF/LR, 743 bp), lane 3 (*ompR*-LF/SR, 2253 bp), lane 4 (Δ*ompR*-LF/SR, 1533 bp), lane 5 (*ompR*-LF/LR, 3180 bp), lane 6 (Δ*ompR*-LF/LR, 2460 bp), lane 7 (*ompR*-SF/SR, 1536 bp), and lane 8 (Δ*ompR*-SF/SR, 816 bp).

**Figure 2:**
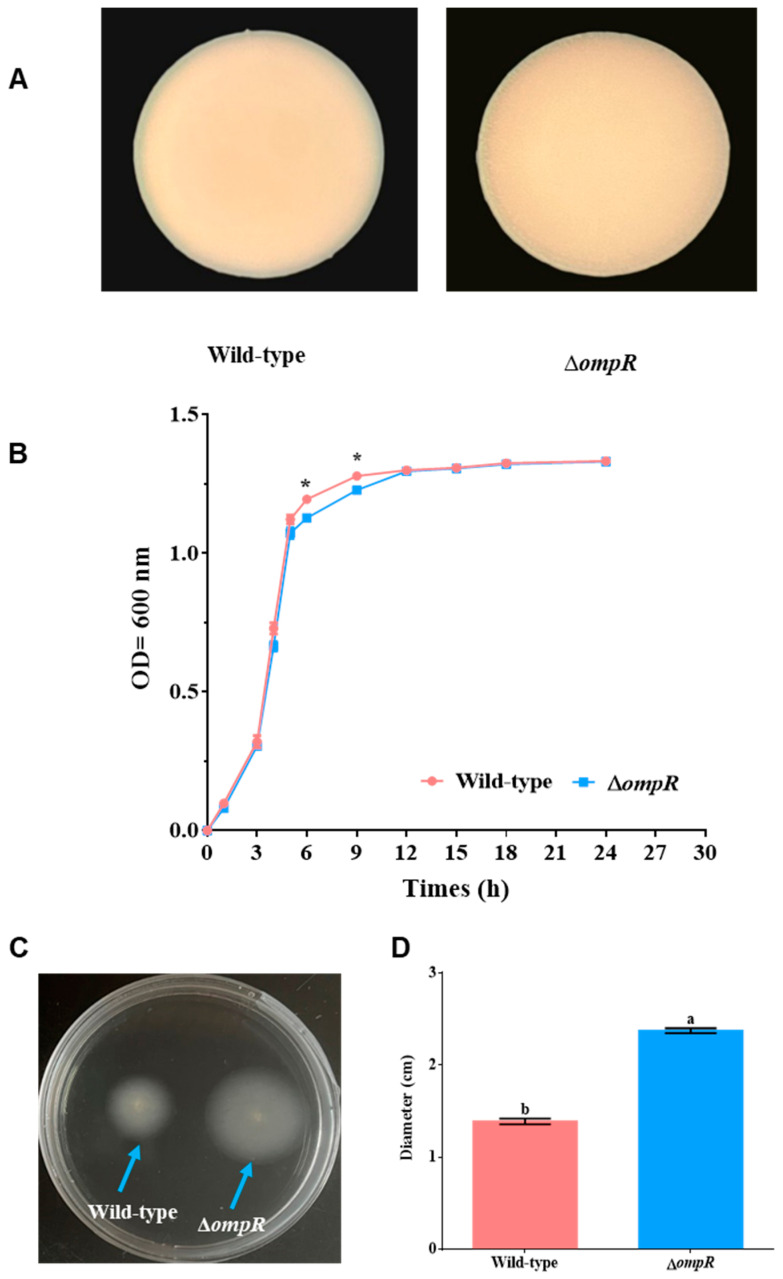
The growth ability, colony morphology (×50), and bacterial motility of wild-type and Δ*ompR* strains. (**A**) Colony morphology of wild-type and Δ*ompR* strains; (**B**) growth curve of wild-type and Δ*ompR* strains (n = 3); (**C**) swimming motility of wild-type and Δ*ompR* strains; and (**D**) migration zone diameter of wild-type and Δ*ompR* strains. Asterisks indicate significant differences (*p* < 0.05); different letters indicate significant differences (*p* < 0.05).

The gene *ompR* was identified by genome annotation from the complete genome of *P. marina*. The in-frame deletion mutant of *ompR* of *P. marina* was constructed, and the coding region was deleted by 721 bp (Figure 1). The biological characteristics of the mutant strain with the deletion of the *ompR* gene were explored (Figure 2). The single colony of wild-type and mutant strains was regular and round with a smooth surface, and there was no obvious change between them (Figure 2A). Wild-type and mutant strains grew rapidly from 3 to 6 h and gradually slowed down after 12 h. During 6 to 9 h, the growth ability of *P. marina* was significantly faster than the Δ*ompR* strain (*p* < 0.05, Figure 2B). 

In comparison with *P. marina*, the Δ*ompR* strain showed enhanced motility, which had a greater range of motion (Figure 2C). The knockout of the *ompR* gene affected the motility of strains, and the diameter of the formed motile migration zone also increased by 70.93% in contrast to *P. marina* (*p* < 0.05, Figure 2D).

**Figure 3:**
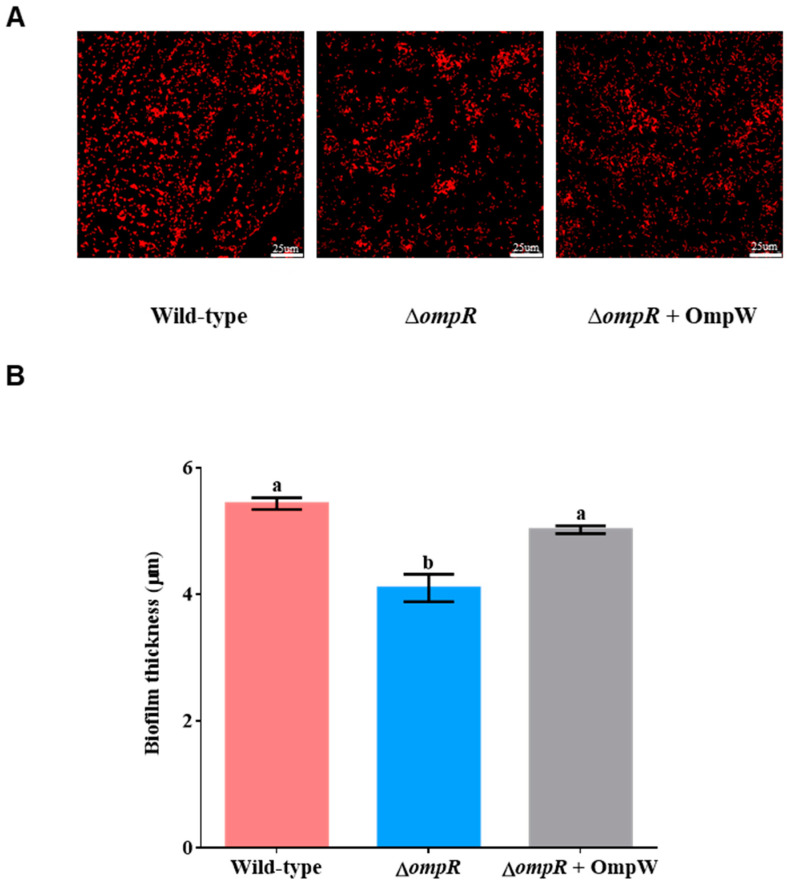
Biofilm formation and thickness of wild-type and Δ*ompR* strains. (**A**) The CLSM images of biofilms formed by the wild-type strain, Δ*ompR* strain, and the Δ*ompR* strain co-cultured with 10 mg L^−1^ recombinant OmpW protein; and (**B**) statistical analysis of strain biofilm thicknesses (n = 9). Different letters indicate significant differences (*p* < 0.05).

CLSM scanning images showed that there were more bacteria clustered in the *P. marina* biofilms, and the bacterial distribution of the mutant biofilms was significantly loose and reduced compared to the *P. marina* biofilms (Figure 3A). The biofilm thickness of the Δ*ompR* strain decreased by 24.54% compared to *P. marina* biofilms (*p* < 0.05, Figure 3B), so the mutant strain had a weaker biofilm formation ability. In the biofilms formed by the Δ*ompR* strain co-cultured with 10 mg L^−1^ recombinant OmpW protein, the number of bacteria on the biofilms increased, and the distribution and aggregation of bacteria on the biofilm surface were similar to the wild-type biofilms (Figure 3A), with the biofilm thickness increasing significantly (*p* < 0.05, Figure 3B), and reaching the levels of *P. marina*.

### 2.2. Knockout of ompR Gene Inhibited Mussel Settlement

**Figure 4:**
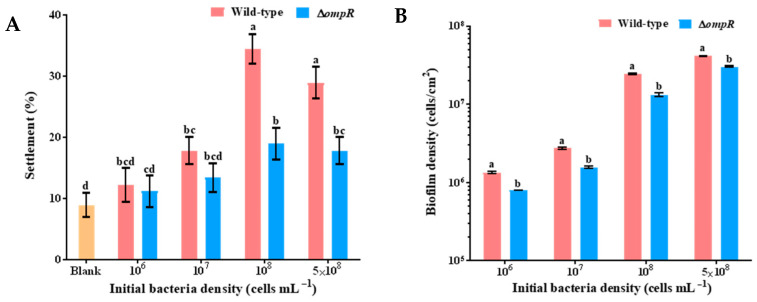
The effect of wild-type biofilms and Δ*ompR* biofilms on mussel settlement and the biofilm density with different initial bacterial densities. (**A**) Inducing activities of biofilms formed by tested strains on mussel settlement; and (**B**) biofilm density on glass slips with different initial bacterial densities of tested strains. Different letters indicate significant differences (*p* < 0.05).

By comparing with *P. marina*, the biofilm formation ability of the Δ*ompR* strain and the inducing activity to the plantigrade settlement of *M. coruscus* were investigated. It was revealed that the deletion of the *ompR* gene significantly reduced the settlement rate of plantigrades (*p* < 0.05), and the settlement rate decreased most significantly when the bacterial density was 1 × 10^8^ cells mL^−1^. Compared with the *P. marina* biofilms, the induction activity of the mutant biofilms decreased by 45.16% (Figure 4A). In addition, at different initial bacterial densities, the Δ*ompR* biofilm densities all decreased significantly (*p* < 0.05, Figure 4B). 

### 2.3. CLSM Images of Biofilms

**Figure 5:**
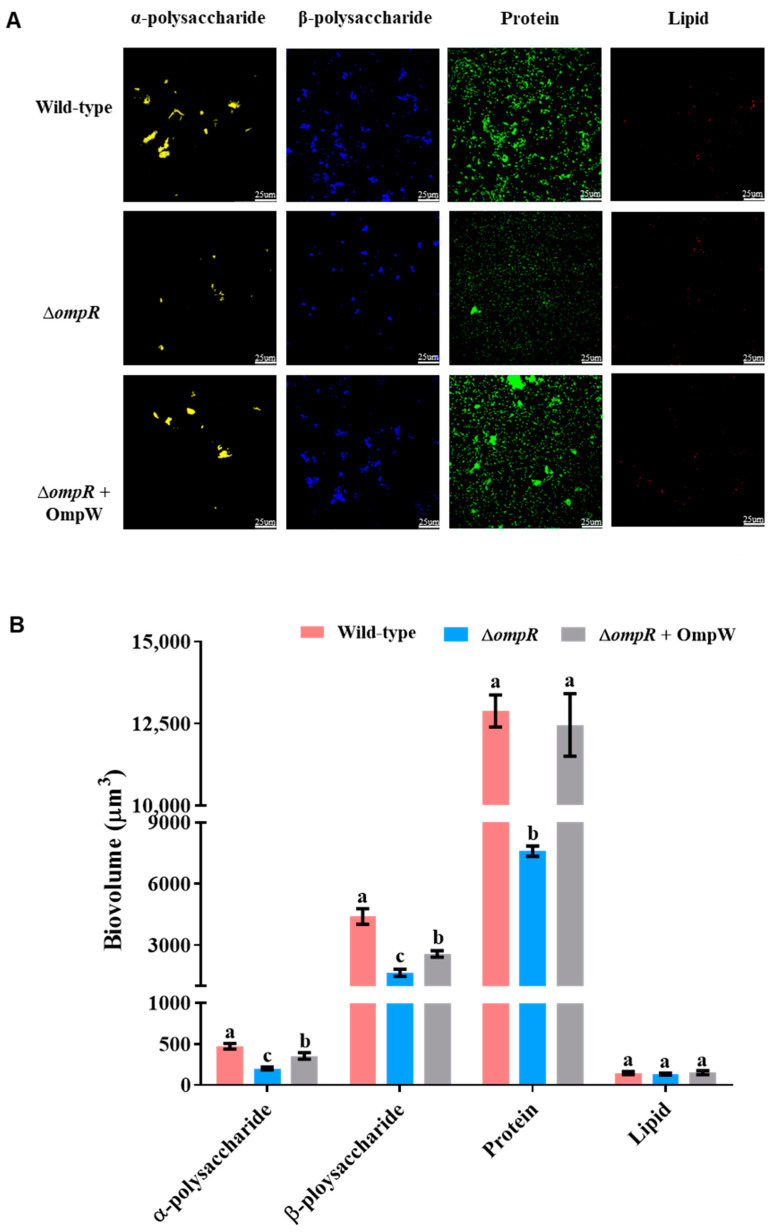
The effect of *ompR* gene deletion on the content of EPS in biofilms. (**A**) The CLSM images of EPS in wild-type biofilms, Δ*ompR* biofilms, and biofilms formed by Δ*ompR* co-cultured with 10 mg L^−1^ recombinant OmpW protein; and (**B**) biovolume analysis of EPS in biofilms. Different letters indicate significant differences (*p* < 0.05).

To further analyze the effect of *ompR* deletion on biofilm formation ability, the CLSM images showed that the extracellular α-polysaccharide, β-polysaccharide, and protein contents were changed significantly in wild-type strains and mutant strains. There was no significant difference in lipid content (Figure 5A). Statistical analysis of the data of the EPS also showed a trend consistent with the results of CLSM scanning. The levels of α-polysaccharide, β-polysaccharide, and protein present in the Δ*ompR* biofilms were lower than those in *P. marina* biofilms by 57.27%, 62.63%, and 41.09%, respectively (*p* < 0.05, Figure 5B).

The extracellular α-polysaccharide, β-polysaccharide and protein levels of Δ*ompR* biofilms (with 10 mg L^−1^ OmpW) and wild-type biofilms were significantly higher than Δ*ompR* biofilms; however, the biovolumes of α-polysaccharide and β-polysaccharide of the Δ*ompR* biofilms with 10 mg L^−1^ OmpW were still lower than the wild-type biofilms (Figure 5A), which reached 75.18% (*p* < 0.05) and 58.35% (*p* < 0.05) of the wild-type biofilms levels, respectively. The exolipid contents did not change significantly among the three groups (Figure 5B).

### 2.4. Relative Expression of ompR, envZ, and ompW Gene and C-di-GMP Content

**Figure 6:**
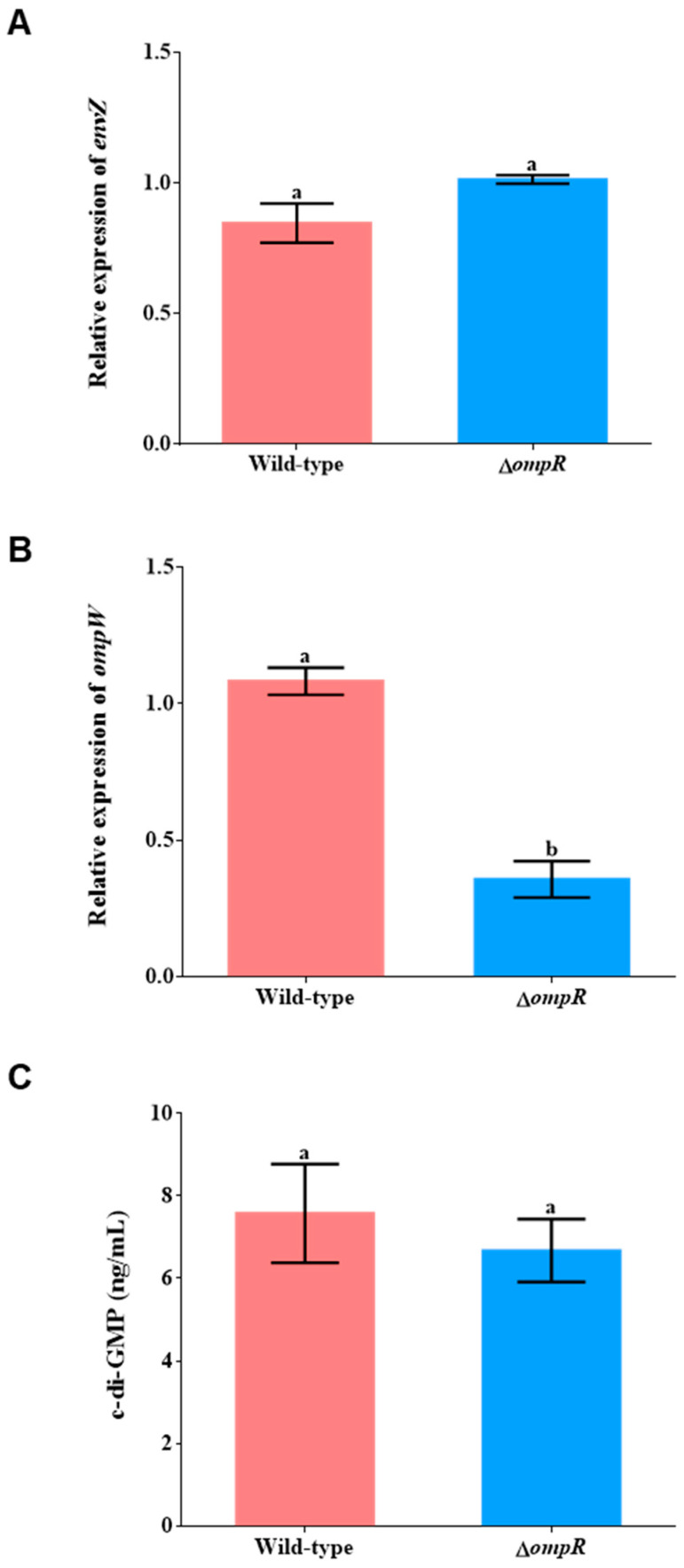
The difference in *envZ* (**A**) and *ompW* (**B**) gene expression, and c-di-GMP concentration (**C**) of experimental strains. Different letters indicate significant differences (*p* < 0.05).

After the *ompR* gene was knocked out, the relative expression of the *envZ* gene between the wild-type biofilms and Δ*ompR* biofilms had no significant difference (*p* > 0.05, Figure 6A). The expression results of the *ompW* gene in the biofilms of the wild-type and mutant strains showed that the *ompW* gene expressed in both strains; however, the expression level of the *ompW* gene in the Δ*ompR* biofilms was significantly lower (67.05% lower) than in the *P. marina* biofilms (*p* < 0.05, Figure 6B). No significant difference in the bacterial c-di-GMP concentrations between the two strains was found (*p* > 0.05, Figure 6C).

### 2.5. Settlement-Inducing Activity of ΔompR Biofilms Formed by the ΔompR Strain Co-Cultured with Recombinant OmpW Protein 

**Figure 7:**
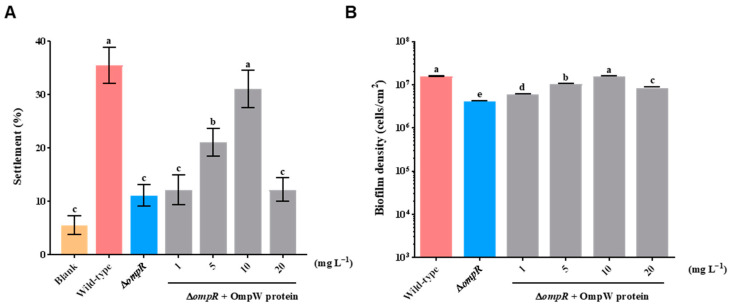
Effect on the inducing activity and biofilm density of Δ*ompR* biofilms after adding recombinant OmpW protein. (**A**) The effect of the inducing activities of biofilms formed by the Δ*ompR* strain on the plantigrade settlement after the addition of different concentrations of OmpW; and (**B**) the biofilm density of the Δ*ompR* biofilms on glass slips after the addition of OmpW. Different letters indicate significant differences (*p* < 0.05).

Four concentrations of 1, 5, 10, and 20 mg L^−1^ recombinant outer membrane protein OmpW were added into Δ*ompR* bacterial solution to form biofilms, respectively. The addition of 10 mg L^−1^ recombinant OmpW significantly improved the induction activity of the mutant biofilms and restored them to the level of wild-type biofilms (*p* > 0.05, Figure 7A). The biofilm bacterial density was increased significantly than the mutant biofilms (*p* < 0.05, Figure 7B) compared with the biofilms formed by Δ*ompR* strains co-cultured with different concentrations of the recombinant OmpW protein.

## 3. Discussion

Many studies have shown that biofilms can affect the settlement of benthic invertebrates, such as *Hydroides elegans* and *Mytilus galloprovincialis* [39,40]. Signaling molecules released by biofilms to induce the settlement of benthic animals are related to EPS; however, the molecular basis of EPS regulation is unclear. Here, our investigation revealed for the first time that the deletion of the *ompR* gene could downregulate the outer membrane protein gene *ompW* and reduce the content of exopolysaccharides, thus effectively decreasing the settlement of mussels.

The deletion of the *ompR* gene enhanced bacterial motility and inhibited biofilm formation in this study. In *E. coli,* OmpR plays a negative regulatory role in the expression of the flagella *flhDC* gene [41]. In *Xenorhabdus nematophila*, deletion of the *ompR* gene can significantly promote bacterial swimming ability [42]. Furthermore, motility is a major dynamic feature [43]. The slow motility properties stabilize the bacterial adhesion process in *P. marina* [6]. In this study, the lower biofilm density and thickness may result from the deletion of the *ompR* gene, which improved the bacterial motility, resulting in the reduction in cell aggregation and poor biofilm formation ability. 

In this study, the knockout of the *ompR* gene in *P. marina* caused a distinct decrease in the expression level of the *ompW* gene, accompanied by the downregulation of the content of exopolysaccharides. In *Shewanella oneidensis*, it was found that the EnvZ/OmpR-dependent regulation of the porin gene almost completely exists in *ompR*, and only partially in *envZ* [44]. Similarly, our present study found that there was no significant difference in the expression level of the *envZ* gene when deleting the *ompR* gene. Thus, the *ompR* gene regulates the change in the exopolysaccharide content of the biofilm, which may be not through the *envZ* gene.

In some bacteria, *ompR* has been found to regulate the outer membrane proteins [45,46,47]. EnvZ/OmpR can induce the expression of multiple OMP genes, including *ompW* [48]. Our studies suggested that the deletion of the *ompR* gene in *P. marina* caused the downregulation of *ompW* gene expression and the decrease in exopolysaccharides in the mutant biofilms. Furthermore, the results of the OmpW replenishment experiment showed that the addition of the exogenous protein OmpW increased the content of polysaccharides in the biofilms formed by the mutant bacteria. Therefore, it is speculated that the content change in exopolysaccharides in bacterial biofilms is related to the OmpW protein. However, whether extracellularly added OmpW has similar effects to the naturally expressed OmpW localized in the membrane is unknown. In addition, how extracellularly added protein participates in the secretion of exopolysaccharides remains unclear. 

The results of this research indicated that the knockout of the *ompR* gene did not change the content of bacterial c-di-GMP. In *Klebsiella pneumoniae*, the *ompR* gene was found to be the key regulator of the c-di-GMP signal pathway and in affecting the biofilm biovolume [49]. Under the condition of the low osmotic pressure of *Yersinia enterocolitica*, the deletion of the *ompR* gene brought about fewer c-di-GMP produced by bacteria [50]. This means that the regulatory relationship between the *ompR* gene and the second messenger is different in diverse bacteria. 

Bacterial exopolysaccharides are key inducers for the larval settlement of benthic invertebrates [6,35,51]. In this study, the inactivation of the *ompR* gene was the cause of lower extracellular α-polysaccharide and β-polysaccharide levels in Δ*ompR* biofilms. In addition, higher exopolysaccharide levels of biofilms formed by the Δ*ompR* strain co-cultured with recombinant OmpW protein were found. Simultaneously, the induced activity of the Δ*ompR* biofilms was upregulated and recovered to the level of *P. marina* biofilms after adding the recombinant protein OmpW. Moreover, after deleting the *ompR* gene, the expression level of the *ompW* gene decreased, and the content of the exoprotein decreased, suggesting that the expression of the OmpW protein may be reduced. Therefore, it is speculated the reduction in the OmpW protein of Δ*ompR* biofilms reduces the content of exopolysaccharides, thereby inhibiting the settlement of mussels.

Notably, after OmpW supplementation, the biofilm-forming capacity of the Δ*ompR* biofilms increased to the levels of the wild-type biofilms, and the exoprotein content of the Δ*ompR* biofilms recovered to the same levels as the wild-type biofilms. However, the exopolysaccharide content of the reconstituted biofilms was significantly higher than that of the mutant biofilms, but still less than the *P. marina* biofilms. Furthermore, the types and components of exopolysaccharides affected by the exogenous protein addition need further study. On the other hand, the present study did not conduct the complementation test of the *ompR* gene back into the deletion strain. Thus, whether a complementation test of the *ompR* gene into the mutant can restore the biofilm-forming capacity and biofilm-inducing activity remains unclear and needs further bioassays. 

## 4. Materials and Methods

### 4.1. Mussel Plantigrade Cultivation and Recombinant Outer Membrane Protein

The mussel plantigrades tested in the investigation were purchased from the Zhoushan coast (122°75′ E; 30°71′ N). These plantigrades were used after being cultured in naturally filtered seawater for a week. The plantigrades (shell length: 0.56 ± 0.03 mm, shell height: 0.38 ± 0.02 mm) were selected and applied to subsequent settlement experiments. Recombinant outer membrane protein OmpW was obtained from Hangzhou Hua’an Biotechnology Co., Ltd. (Hangzhou, China).

### 4.2. Strains and Plasmids 

*P. marina* was isolated from the natural biofilms. *P. marina* and Δ*ompR* were both cultivated in Zobell 2216E medium at 25 °C, and the *Escherichia coli* WM3064 was cultured with 0.3 mM 2,6-diamino-pimelic acid in Luria–Bertani (LB) medium (Sigma, St. Louis, MO, USA) at 37 °C [30,52,53]. Fifty μg mL^−1^ kanamycins and 25 μg mL^−1^ erythromycin were added in the construction of the mutant strain to maintain the resistance. The specific information is shown in Table 1.

### 4.3. Construction of ΔompR Strain

The Δ*ompR* strain was constructed, as previously described [35,53]. The primers used in the experiment were designed according to the *ompR* gene of *P. marina*. Finally, the Δ*ompR* mutant was tested using *ompR*-SF/*ompR*-LR, *ompR*-LF/*ompR*-SR, *ompR*-SF/*ompR*-SR, and *ompR*-LF/*ompR*-LR. Table 1 exhibits the primers used to amplify the upstream and downstream of the target fragment of the *ompR* gene.

### 4.4. Colony Morphology and Growth Ability Determination

The bacteria were incubated at a constant temperature of 25 °C at 200 r/min and then spread on 2216E solid medium after gradient dilution for 5–7 days before filming. The OD600 absorbance of *P. marina* and Δ*ompR* strains were measured using a spectrophotometer at 1, 3, 4, 5, 6, 9, 12, 15, 18, and 24 h after culture with 2216E liquid medium at 25 °C (200 r/min).

### 4.5. Swimming Motility and Migration Zone Diameter

After the tested strains were cultured for 16–18 h, 1 μL of the *P. marina* and Δ*ompR* bacterial solutions were dropped vertically on the swimming medium. After incubation at 25 °C for 16–18 h, the swimming trajectory was observed. The diameter of the migration zone diameter was determined by setting up 3 groups with 10 parallels in each group.

### 4.6. The Formation of Biofilms

After being cultured for 16–18 h, the cell precipitations of the *P. marina* and Δ*ompR* strains were obtained after centrifugation at 3500 r/min for 15 min, being blown and cleaned in autoclaved filtered seawater (AFSW) 3 times, and then adjusted to a volume of 50 mL. The initial bacterial densities were diluted to 1 × 10^6^, 1 × 10^7^, 1 × 10^8^, and 5 × 10^8^ cells mL^−1^, and the corresponding bacterial solution and AFSW were added to each glass Petri dish. All dishes were loaded with sterilized slides, shielded from light, and stored at 18 °C for 48 h to form biofilms.

The recombinant protein OmpW was added to the bacteria solution (1 × 10^8^ cells mL^−1^) to form biofilms. The concentration gradient of OmpW was set at 0, 1, 5, 10, and 20 mg L^−1^.

### 4.7. The Thickness of Biofilms

Tested biofilms were soaked in formalin for half an hour in advance and then rinsed with 0.9% normal saline. Propidium iodide solution was added to the biofilm for 20 min, and the dye was removed with normal saline. To measure the biofilm thickness, the images of confocal laser scanning microscopy (CLSM) were collected [6,31]. Each biofilm had three biological replicates, and ten domains were randomly selected for each biofilm.

### 4.8. The Bacterial Density of Biofilms

After being fixed in 5% formaldehyde solution for more than 24 h, the tested biofilms were soaked in acridine orange solution for 5 min in the dark, then the dye was dried and the samples sealed. Each biofilm test contained three biological replicates, and ten fields were randomly selected from each biofilm to count the cell density using a fluorescence microscope at 1000×.

### 4.9. Settlement Bioassay

A glass slide containing biofilm was placed in a Petri dish to which 20 mL AFSW and 10 plantigrades were added into one dish, and 9 replicates were set per group. The samples were then incubated at 18 °C in the dark, and the settlement of the plantigrades was observed at 48 h.

### 4.10. CLSM of Biofilms

Biofilms were stained with 50–70 μL of the corresponding dyes. α-polysaccharide was stained with ConA-TMR, β-polysaccharide was stained with Calcofluor White stain, DiD Oil was used to stain lipids, and FITC was used to stain protein. After 25 min, the floating color on the surface was washed off and the images were captured under a Leica confocal microscope, as previously described in the methods of the literature [6,31]. Recombinant OmpW protein was added at a concentration of 10 mg L^−1^ to the Δ*ompR* bacteria solution (1 × 10^8^ cells mL^−1^) to form biofilms for CLSM images. 

### 4.11. RT-qPCR of Biofilms and Bacterial C-di-GMP

The bacterial RNA of wild-type and Δ*ompR* biofilms was extracted using Trizol reagent, and the cDNA was then obtained by reverse transcription and stored at −20 °C for later use. According to the CDS sequences of the *envZ* and *ompW* genes, the corresponding *envZ* -RT-F/R and *ompW*-RT-F/R primers were designed using Primer 5.0 (Table 2), and *gyrB* were used as the internal control gene. The qPCR experiment was conducted with the Cham Q TM Universal SYBR^®^ qPCR Master Mix Kit. The relative expressions of the *envZ* and *ompW* genes in biofilms formed by wild-type and mutant strains were analyzed using the 2^−ΔΔCT^ method and shown as mean ± standard deviation. 

The determination of c-di-GMP content was conducted per the previously described method [6].

### 4.12. Data Analysis

Image J software (Wayne Rasband, National Institutes of Health, Bethesda, MD, USA, version 1.52a) was utilized to analyze the CLSM images of EPS on biofilms and SPSS 25.0 software was used for differential analysis.

## 5. Conclusions

Knocking out the *ompR* gene upregulated the bacterial motility and downregulated the biofilm-inducing and forming capacity, accompanied by a decrease in the outer membrane protein *ompW* gene expression levels and exopolysaccharides, thus inhibiting the settlement of *M. coruscus*. In addition, OmpW protein supplementation increased the content of exopolysaccharides and promoted mussel settlement. This study is positively significant in exploring the interaction between outer membrane proteins and settlement and provides new insight into the interaction between biofilms and hosts.

## Figures and Tables

**Table 1:** Strains, plasmids, and primers were used in this study.

	Bacterial Strains/Plasmids/Primers	Relevant Features	Source
Strains	*E. coli* WM3064	RP4 (tra) in the chromosome, DAP	[52]
	*P. marina* ECSMB14103	Wild-type	[5,6]
	Δ*ompR*	The deletion of the *ompR* mutant strain	This study
Plasmids	pK18mobsacB-ery	pK18mobsacB with erythromycin resistance	[54]
	pK18mobsacB-ery-*ompR*	Recombinant plasmid used to knock out *ompR* genes	This study
Primers	*ompR*-UF	CGCGGATCCCGAATGCGAGTAAGTGGTGT	This study
	*ompR*-UR	CCGCTCGAGTTCCTGACGGTGAAAAGTAG	This study
	*ompR*-DF	CCGCTCGAGTTTGTCGTTTCGTGTCCCAT	This study
	*ompR*-DR	ACGCGTCGACCCTTTAGGGAGTGGTTGAGC	This study
	*ompR*-SF	TACCCTGAAAGCGGAATT	This study
	*ompR*-SR	GCGAACACGTCGGTCTAT	This study
	*ompR*-LF	GGTTCAAATACCGACTCTA	This study
	*ompR*-LR	GATTGTTGTTTCGTGCTGT	This study

**Table 2:** The primer sequences used in this study.

Primer Name	Primer Sequence (5′-3′)	Usage
envZ-RT-F	GGTATAGCGGCCCTTGAAAA	RT-qPCR
envZ-RT-R	GATACTCGTCTTGGTCGCTC	RT-qPCR
ompW-RT-F	ATTGATTGCTGCTACGCC	RT-qPCR
ompW-RT-R	CTGTGCCACTAACGAGGG	RT-qPCR
gyrB-RT-F	GCAGCCGAAACGCCTTCTTCT	RT-qPCR
gyrB-RT-R	CCGATGATGGCACAGGCTTACA	RT-qPCR

## Data Availability

The data of this study are available in this article.
